# HIV peripheral neuropathy‐related degeneration of white matter tracts to sensorimotor cortex

**DOI:** 10.1007/s13365-022-01051-w

**Published:** 2022-10-07

**Authors:** Sara H. Timtim, Alan N. Simmons, Chelsea Hays, Irina Strigo, Scott Sorg, Ronald Ellis, John R. Keltner

**Affiliations:** 1 UCSD, University of California San Diego School of Medicine, San Diego, CA USA; 2 UCSF, University of California San Francisco School of Medicine, San Francisco, CA USA

**Keywords:** HIV neuropathy, Distal sensory polyneuropathy, Diffusion tensor imaging (DTI), Cortical atrophy

## Abstract

Human immunodeficiency virus-associated distal sensory polyneuropathy (HIV-DSP) affects up to 50% of people with HIV and is associated with depression, unemployment, and generally worsened quality of life. Previous work on the cortical mechanism of HIV neuropathy found decreased gray matter volume in the bilateral midbrain, thalamus, and posterior cingulate cortex, but structural connectivity in this context remains under-studied. Here we examine alterations in white matter microstructure using diffusion imaging, hypothesizing that cortical white matter degeneration would be observed in continuation of the peripheral white matter atrophy previously observed in HIV-DSP. Male HIV seropositive patients (n = 57) experiencing varying degrees of HIV neuropathy underwent single-shell diffusion tensor imaging with 51 sampling directions. The scans were pooled using tractography and connectometry to create a quantitative map of white matter tract integrity, measured in generalized fractional anisotropy (GFA). The relationship between GFA and neuropathy severity was evaluated with linear regression. Correction for multiple comparisons was done using false discovery rate (FDR), a statistical method commonly used in genomics and imaging to minimize false positives when thousands of individual comparisons are made. Neuropathy severity was associated with decreased GFA along thalamocortical radiations leading along the lateral thalamus to sensorimotor cortex, with r = -0.405 (p < 0.001; FDR), as well as with the superior bilateral cingulum (r = -0.346 (p < 0.05; FDR)). Among a population of HIV neuropathy patients, greater neuropathy severity was correlated with lower white matter integrity running from midbrain to somatosensory cortex. This suggests ascending deafferentation extending from damaged peripheral nerves further downstream than seen previously, into the axons of third-order neurons. There is also evidence of cingulum degeneration, implying some more complex mechanism beyond the ascending atrophy observed here.

## Introduction

Peripheral neuropathy associated with human immunodeficiency virus (HIV) affects as many as 50% of HIV-infected individuals, often causing pain that is one of the most common and treatment-resistant neurologic complications of HIV (Schütz and Robinson-Papp 2013). The most common neuropathy pattern is a distal, symmetric, sensory polyneuropathy with indolent course, featuring some combination of paresthesia, sensory loss, and/or pain—the latter affecting up to 40% of patients (Evans et al. 2011). HIV-DSP is significantly associated with depression, unemployment, and overall worsened quality of life, especially in those whose neuropathy pattern includes pain (Ellis et al. 2010). HIV-DSP-associated neuropathic pain typically responds poorly to established chronic pain therapies as well as antiretrovirals (indeed toxic neuropathy caused by antiretroviral exposure is a major variant included in HIV neuropathy) (Jensen et al. 2019; Morgello et al. 2004). While symptom severity is correlated with specific peripheral pathways and markers (e.g., distal epidermal denervation), peripheral mechanisms cannot explain the full range of distressing symptoms of pain and paresthesia (Simpson et al. 2006). Brain-based analyses have helped bridge this gap by improving understanding of central mechanisms contributing to pain and paresthesia symptoms for HIV-DSP (Keltner et al. 2014).

While brain imaging studies of HIV neuropathy are few, findings suggest gray matter loss in the posterior cingulate cortex (PCC), thalamus, and midbrain (Keltner et al. 2017). Insights into peripheral neuropathy brain mechanisms can likely be drawn from other chronic distal symmetric polyneuropathies, such as diabetic neuropathy. In cases of painful diabetic neuropathy, gross anatomical changes have been observed in the sensorimotor cortex, including reduction in gray matter volume of this overall region and expansion of the area representing pain in the lower limbs (Selvarajah et al. 2019). Given that the gross volume changes described previously become discernible only after decades of chronic pain, diffusion tensor imaging (DTI) may detect subtle white matter changes that occur earlier in the disease course or in milder disease, shedding light on specific mechanisms. We aimed to bridge this gap using DTI to investigate whether HIV neuropathy severity was related to changes in white matter connectivity in the brain. Based on prior research, we expected to find evidence of white matter atrophy of higher brain structures (eg second- and third-order neurons) seeming to result from chronic under-stimulation by damaged peripheral nerves as seen in gray matter studies of other peripheral neuropathies (Selvarajah et al. 2018).

Diffusion MRI methods have been useful to non-invasively characterize white matter microstructure in vivo*.* A common metric is fractional anisotropy (FA), which captures how strongly water diffuses unidirectionally, as it would be expected to when constrained within the organized long hydrophobic axon tracts of white matter. FA is limited in its ability to capture some common physiological cases such as where white matter fibers cross-paths, or a single voxel contains partial volumes of different tissues; it also cannot differentiate between increased axon density versus increased myelination. In this study, we used generalized fractional anisotropy (GFA), an extension of FA validated in previous studies to account for some of these complexities (Yeh et al. 2013). Its calculation is based on sampling the diffusion orientation distribution function; as the specific method exceeds the scope of this paper, for details please see reference (Glenn et al. 2015; Tuch 2004). Like FA, GFA can be interpreted as the degree of preferential directional diffusion mobility, with the benefit of capturing more complex diffusion profiles (Glenn et al. 2015).

## Methods

### Participants

Study participants were male HIV-seropositive outpatients experiencing at least two symptoms of distal sensory neuropathy, between the ages of 18 and 70 (mean age 58.2, SD 7.9). Fifty-seven subjects completed the procedures with usable scans (five additional subjects were scanned but excluded due to concerns with image quality). All except three were currently taking HAART, average exposure time 191 months ± 85 months. Neuropathy severity was judged solely on the basis of clinical findings without considering underlying HIV severity quantitatively such as CD4 + count, as this had been examined previously. For full details of exclusion criteria, please see Appendix. Briefly, exclusion criteria were neurological morbidity external to HIV illness, any severe medical condition unrelated to HIV requiring inpatient treatment, or frequent medical follow-ups including but not limited to: unstable hypertension, unstable angina, unstable diabetes mellitus, unstable cardiac arrhythmias, transient ischemic attacks, severe coronary artery disease, severe peripheral vascular disease, severe hepato-gastro-intestinal disease, severe infectious disease, central nervous system neoplasms, or substance abuse within the last 30 days. The study was approved by the UCSD Human Research Protections Program, and all participants signed an informed consent document (Table 1).

**Table 1:** Participant demographics

	**Mean**	**SD**
Age (years)	58.2	7.87
Education (years)	14.8	2.96
Total neuropathy severity	8.94	3.87
Paresthesia severity	1.61	0.818
MOS physical function*	65.5	22.2
Race		
White	46 (80.7%)	
Black	6 (10.5%)	
Hispanic/mixed	5 (8.8%)	

^*^MOS (Medical Outcomes Study) (Tarlov et al. 1989) scoring: 0 = max disability, 100 = no disability

### Clinical procedure and metrics

Patients qualified as having peripheral neuropathy sufficient for inclusion in the study if they displayed at least two clinical signs (eg reduced vibration sensation, sharps sensation, or deep-tendon reflexes, and a specific pattern of abnormal tingling or pricking sensations in the distal bilateral lower extremities (Ellis et al. 2010), as evaluated by a study physician. Clinical severity was quantified using Total Neuropathy Score (TNS), a tool validated with high inter- and intrarater reliability to measure neuropathy in various settings, including diabetic, chemotherapy-induced, and recently HIV neuropathy (Cavaletti et al. 2007). TNS combines a clinician assessment with patient self-report of sensory and motor symptoms. The clinician assessment includes deep tendon reflexes, manual testing of distal muscle strength, sharp sensitivity using a pin, number of autonomic symptoms (e.g., sweating, orthostatic intolerance, pupillomotor dysfunction, etc.), and vibration sensitivity using a graduated Rydel–Seiffer tuning fork. Higher scores indicate worse neuropathy, ranging from 0–44 points with ≥ 5 generally deemed clinically significant (Smith et al. 2008).

### MRI acquisition

Diffusion and T1 scans were obtained with a 3 T GE Discovery MR750 scanner. 51 diffusion directions were collected at b-value of 1000 s/mm^2^ in addition to a B0 image. Repetition time (TR) was 9.700 s, echo time (TE) was 81.1 ms, flip angle = 90°. Acquisition matrix size was 96 × 96, and reconstruction matrix size was 128 × 128; 51 slices with a thickness of 2.5 mm were acquired for each direction. Two field maps were also acquired to correct for susceptibility-induced distortions T1-weighted structural image was acquired with the following parameters: 172 sagittal slices, TR = 8.132 ms, TE = 3.192 ms, flip angle = 8°, slice thickness = 1.2 mm, reconstruction matrix PE = 256, FOV = 256 × 256 mm.

### Diffusion anisotropy data processing

Data preprocessing was performed with tools from the Oxford Centre for Functional Magnetic Resonance Imaging of the Brain (FMRIB) Software Library (FSL) (Jenkinson et al. 2012). It included corrections for head motion, eddy currents, and susceptibility-induced distortions, and voxels containing non-brain tissue were masked (Andersson et al. 2003; Smith et al. 2004). Data were reconstructed in the DSI Studio software package using Q-Space diffeomorphic reconstruction (QSDR), a rotational reconstruction that calculates the orientational distribution of the density of diffusing water (Yeh and Tseng 2011). Fidelity of this reconstruction was checked by calculating correlation between each subject’s warped image and the standardized map image (MNI space), yielding R^2^ values all in the 0.70–0.90 range, which indicates good registration accuracy.

### Tractography and connectometry

After pre-processing, tractography and connectometry were performed with DSI Studio, which uses a deterministic fiber-tracking algorithm with quantitative anisotropy (QA) as the termination index (Yeh et al. 2013). Group connectometry, which aggregates the scans of all our subjects, was used to capture differences between individuals. It is an atlas-based analysis method that tracks association/correlation along major fiber pathways, and statistically tests the findings using false discovery rate (Yeh et al. 2016). Multiple regression modeling of total neuropathy score vs GFA was performed, using age as a covariate. The analysis was performed at a whole-brain level with T threshold of 3 (p < 0.01), and length threshold of 40 mm was used to select tracks. To assess statistical significance and correct for multiple comparisons, we estimated the false discovery rate (FDR), applying 10,000 random permutations to obtain a null distribution of track lengths, i.e., distribution if the associations we found happened by chance (Nichols and Holmes 2002).

## Results

### Connectometry results and clustering

**Fig. 1:**
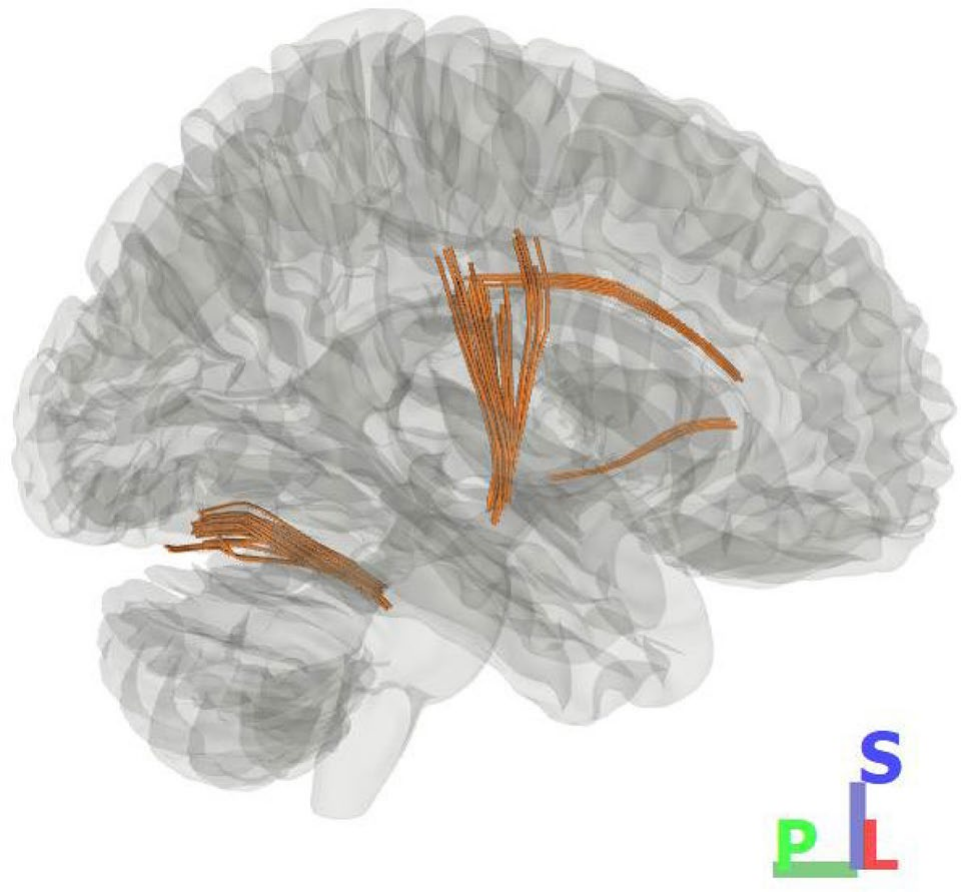
Main Effects. This initial connectometry visualization generated using DSI Studio shows the tracts of significant negative correlation between white matter integrity (GFA) and neuropathy severity (Total Neuropathy Score). Shown from R sagittal view to highlight the primary tract of interest, which is pictured at center, running superior–inferior. For simplicity, only the most significant tracts are shown (see Fig. 3 for full detail)

**Fig. 2:**
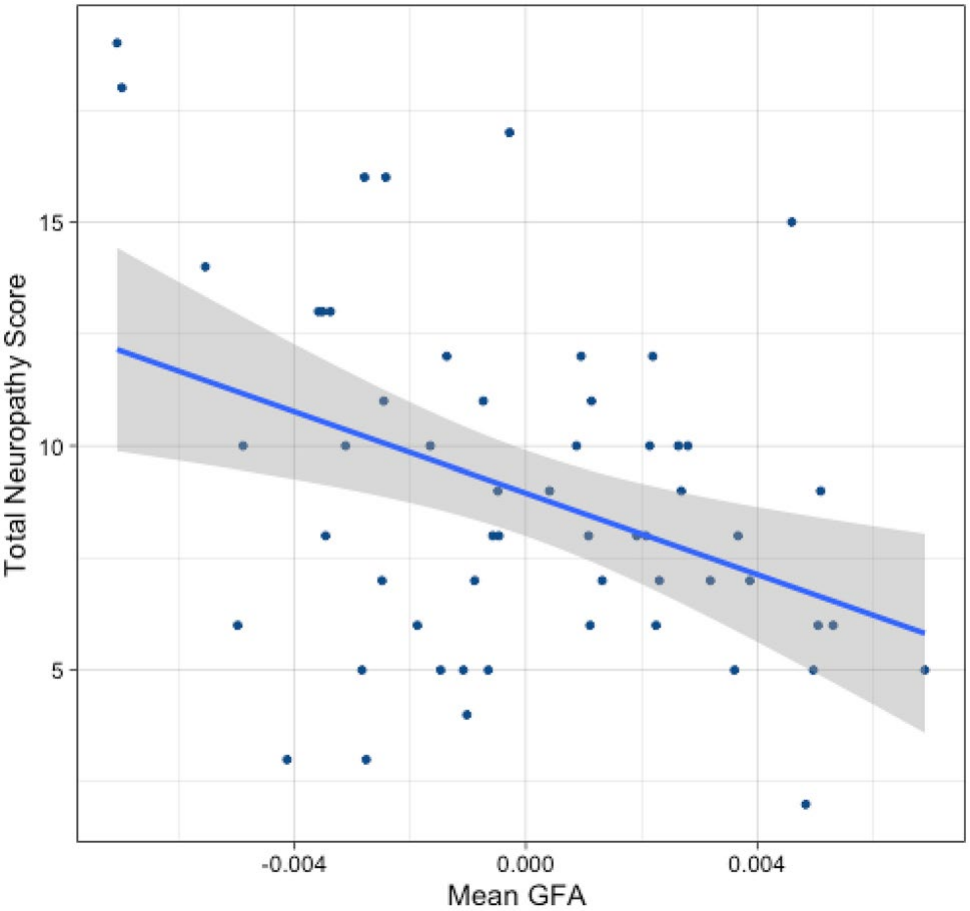
Correlation of neuropathy severity with GFA in the primary tract of interest (after controlling for age) r = -0.405

**Fig. 3:**
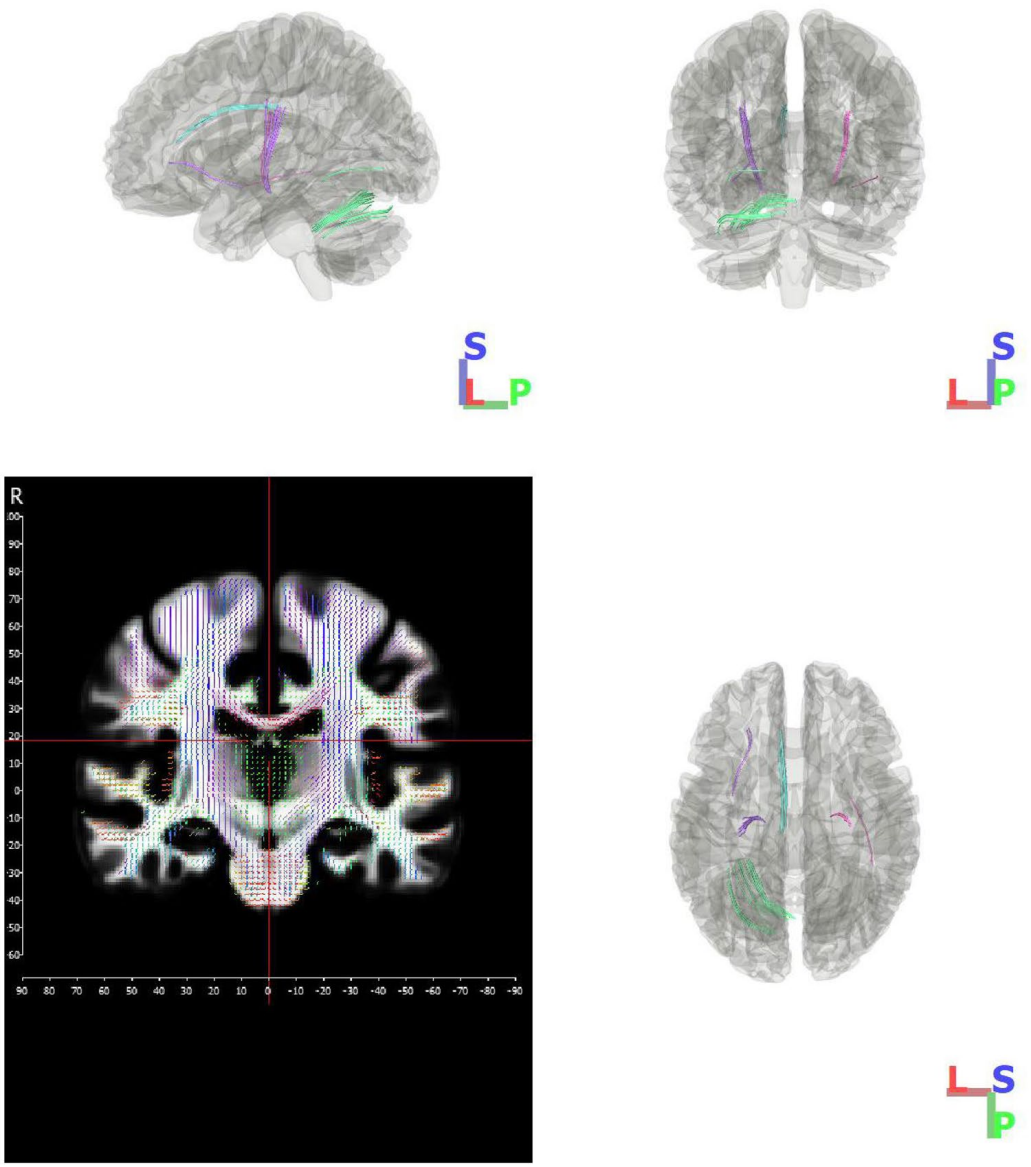
Major tracts of negative correlation which were distinguished from each other using k-means clustering. The primary tract of interest is pictured in pink and purple, and the cingulum cluster is pictured in cyan. Bottom left figure shows orientation distribution

DSI studio’s group connectometry method yields a whole-brain map (overview visual in Fig. 1), without differentiating explicitly between anatomically known/named white matter tracts. The next step was to differentiate these major white matter tracts, enabling further analysis on specific tracts including calculating a mean GFA of each tract. To this end (partitioning the significant pathways into anatomically recognizable and distinct white matter tracts), we used k-means clustering. This clustering yielded a single GFA value for each tract of interest, which could then be used to calculate each tract’s correlation with demographics metrics. A four-cluster solution (k = 4) generated the most anatomically meaningful tracts, and for visual reference, an overview of these four clusters is found in Figs. 2 and 3 (Table 2).

**Table 2:** Sample descriptives

**Figure color**	**Location**	**# of tracts**	**Tract length mean (mm)**	**Tracts volume (mm**^**3**^**)**	**GFA mean**
Cyan	Cingulum L	293	44.42 (4.92)	5280	0.12 (0.03)
Pink	Thalamocortical radiations R	288	48.90 (2.95)	6176	0.04 (0.00)
Purple	Thalamocortical radiations L	276	42.25 (2.03)	6288	0.03 (0.00)
Green	Cerebellum L	97	42.61 (2.38)	3400	0.12 (0.02)

Data provided as mean (standard deviation)

### Main effects

Higher neuropathy severity was significantly associated with lower GFA bilaterally along thalamocortical radiations (Behrens et al. 2003; Zhang et al. 2010), leading along the lateral thalamus to sensorimotor cortex (p < 0.001; FDR). Correlation with TNS for this main tract was r = -0.405 (see Fig. 2). Visualization that includes the position of the thalamus for reference suggests intersection with the ventral posterolateral nucleus (VPL), the thalamic region which relays ascending sensory fibers of the spinothalamic tract to the primary somatosensory cortex. It is important to note DTI connectometry estimates white matter anatomy based on location and cannot definitively differentiate specific fiber types within.

A secondary finding of lower white matter integrity with greater TNS was also seen in the superior section of the cingulum, bilaterally (p < 0.05; FDR).

A third tract that also appeared to reach the level of significance was found in the cerebellum (included in Figs. 1 and 2 for completeness), but this was not examined further due to its positioning near the edge of the image, where technical limitations of the scan method render findings less reliable. To generate a three-dimensional image, scan samples are taken many times (fifty-one in this study), each time from a slightly different direction, with each direction incrementally improving accuracy. At the center of the target, all of the samples overlap, yielding full accuracy, whereas at the outer edges of the target, the scan angles are such that few samples overlap and coverage is inconsistent. We comment on the finding nevertheless, as there has been some recent evidence of cerebellar vermis volume decline in HIV neuropathy which might be explored in future studies (Zahr et al. 2019).

Summary of mean GFA and physical characteristics of each of the significant tracts delineated during k-clustering, corresponding to those pictured in Fig. 3.

## Discussion

Based on known pathophysiology of damaged peripheral nerves in HIV distal sensory-predominant polyneuropathy, previous work hypothesized that decreased regional brain volumes reflect downstream atrophy resulting from impaired signaling from peripheral nerves or peripheral nerve atrophy. The dorsal root ganglion sensory neurons are pseudo-unipolar, with the central process synapsing on the gracile or cuneate nuclei. These, in turn, project to the thalamus, and from there to the sensory cortex. For the first- and second-order neurons of these pathways, atrophy has previously been observed in HIV neuropathy patients via structural MRI—that is, from the peripheral nervous system (via spinothalamic tracts) up through bilateral midbrain to thalamus (VPL and VPM), with peak thalamic atrophy in a region with reciprocal connections to PCC (Keltner et al. 2020). And, in the subset of individuals with distal neuropathic pain, smaller left ventral PCC sizes were seen (Keltner et al. 2014).

Based on the positioning of the white matter tract degeneration seen here, we suspect a new finding of a similar mechanism, now extending beyond the midbrain/thalamic regions described previously, into third-order neurons projecting from thalamus to the somatosensory cortex. This possible downstream afferent white matter tract atrophy would be consistent with pathophysiology observed in non-HIV peripheral neuropathies (Jaggi and Singh 2011; Marchettini et al. 2006; Eaton et al. 2001; Tesfaye et al. 2016; Navarro et al. 2007). It stands to reason that less prominent atrophy would result further downstream in the generalized cortical structures than would occur in the second-order neurons and structures receiving more direct (and weakened) signaling from damaged distal peripheral nerves (Johansen-Berg et al. 2005). Hence, subtle changes in white matter microstructure are detectable via DTI despite no known gross gray matter changes in the eventual cortical target, i.e., somatosensory cortex.

A previous midbrain finding looks to be part of descending white matter tracts, suggesting a reciprocal mechanism of communication between central and peripheral nerve systems rather than simply ascending (Keltner et al. 2020).

The present study also found decreased integrity of the cingulum. This suggests some disrupted cortical circuitry independent of our hypothesized framework of simple ascending atrophy. It is intriguing because the cingulum functionally connects the PCC and mPFC of the default mode network (DMN) (Heuvel et al. 2008), a pathway which has previously been implicated in work on the psychological component of chronic pain. One study found that in chronic pain patients, the expected task-related deactivation of DMN during attention-requiring tasks was smaller than in healthy controls–that is, DMN was less responsive to normal stimuli (Baliki et al. 2008; Greicius et al. 2009). This paper concluded that chronic pain disrupts the DMN, but, the specific meaning of the finding remains unclear. Another study found similar diminished dynamic DMN deactivation during spontaneous attentional fluctuations away from pain, as well as increased connectivity (both structural and resting-state functional) between DMN and the periaqueductal gray (part of the descending pain modulatory system) with tendency toward mind wandering. This paper concluded that increased DMN engagement reflected mind-wandering that distracts from chronic pain (Kucyi et al. 2013). Hence, while the finding of decreased cingulum integrity and its implications for the default mode network is potentially interesting, its significance remains speculative in nature.

Building on this study, future work would explore the specific contribution of neuropathic pain. The ~ 40% of HIV-DSP patients whose presentation includes pain experience markedly worse prognosis and reduction in quality of life than those without pain, and it remains unknown why some patients are affected but not others. The present study examined neuropathy severity as a general spectrum, without binary group comparison between those with and without pain (which was beyond this study’s statistical power). This conceptualization may obscure some particular effect of pain, and the risk of conflation is reflected in correlation of around 67% between TNS and last-12h neuropathic pain in this sample.

A basic limitation of the study is cross-sectional design, which restricted our ability to draw causal conclusions particularly while attempting to characterize progression of a chronic illness. Resolution was also limited in this study, with 51 sampling directions and single-shell DTI; more advanced acquisition techniques such as HARDI or multi-shell approaches could provide increased accuracy, as could correlation with functional imaging. Acquisition was also done with non-isotropic voxel size, which can be addressed during image processing by resampling as we did here, but generally causes modeling complications compared to isotropic voxels, for example, underestimating FA values measured in regions containing crossing fibers (Oouchi et al. 2007; Jones et al. 2013). As with any tractography study, results at a group level necessarily vary based on the parameters chosen to set significance threshold, including track length and T threshold, with tradeoffs in sensitivity/specificity. Relatively small sample size of n = 57 (though this is somewhat typical for DTI studies) and subjects all being male further limit generalizability.

## Conclusions

Using diffusion imaging to visualize white matter tracts, we observed more severe HIV neuropathy associated with degradation of tracts leading from the midbrain along lateral thalamus toward the sensorimotor cortex. These findings suggest that neural atrophy stemming from peripheral nerve dysfunction may extend further than previously thought, beyond the midbrain and thalamus and up into the cortex. The cingulum, a white matter tract implicated in studies of other chronic pain conditions, with psychiatric implications for individuals’ varying experience of pain, appeared similarly weakened among those with greater neuropathy severity, inviting future exploration of a potentially more complex neural mechanism underlying HIV peripheral neuropathy.

## Appendix

### Supplemental Figures

**Fig. 4** Main tract with relevant structures highlighted for location reference, thalamus (depicted in blue) to sensorimotor cortex (depicted in yellow)

### Exclusion Criteria

1. Neurocognitive morbidity that is external to HIV illness: e.g., chronic renal disease chronic pulmonary disease, autoimmune disorders.
2. Serious co-morbid medical condition unrelated to HIV: current severe medical disorder that requires inpatient treatment or frequent medical follow-ups including but not limited to: unstable hypertension, unstable angina, unstable diabetes mellitus, unstable cardiac arrhythmias, transient ischemic attack, severe coronary artery disease, severe peripheral vascular disease, severe hepato-gastro-intestinal disease, severe infectious disease.
3. Neurological confounds: e.g., head injury with loss of consciousness for greater than 30 min, seizure disorders; CNS neoplasms unrelated to HIV; multiple sclerosis
4. Severe psychiatric disorder that might make participation of the subject problematic or unsafe,
5. Current intoxication or active abuse/dependence within last 30 days
6. Other conditions: Women that are pregnant, breast-feeding or of child-bearing potential who are not practicing reliable contraceptive methods (if uncertain whether a subject might be pregnant, the fMRI scan will only be performed after a negative pregnancy test result obtained).
7. Any metal prosthesis or device, including pacemakers, aneurysm clips, heart/vascular clips, etc.
8. Claustrophobia.
